# The Relationship between Serum Iron and Thyroid Function in the Patients with Type 2 Diabetes

**DOI:** 10.2174/1871530322666220928144548

**Published:** 2023-04-07

**Authors:** Congcong Wang, Song Wen, Xinlu Yuan, Mingyue Zhou, Yanyan Li, Min Gong, Jianlan Jin, Ligang Zhou

**Affiliations:** 1 Department of Endocrinology, Shanghai Pudong Hospital, Fudan University, Shanghai 201399, China;; 2 Clinical Research OB/GYN REI Division, University of California, San Francisco, USA;; 3 Shanghai Key Laboratory of Vascular Lesions Regulation and Remodeling, Shanghai Pudong Hospital, Shanghai, 201399, China

**Keywords:** Type 2 diabetes mellitus, serum iron, thyroid function, free thyroid hormones, total thyroid hormones, thyroid-stimulating hormone

## Abstract

***Purpose*:** Our primary objective in this study is to determine the relationship between serum iron (Fe^3+^) and thyroid functions in type 2 diabetes mellitus (T2DM) patients.

***Materials and Methods*:** Glucose metabolic parameters, trace elements, such as Fe^3+^, and thyroid functions for 1657 type 2 diabetic patients treated at the Shanghai Pudong Hospital's Department of Endocrinology from 2018 to 2021 were assessed.

***Results*:** Variations in free thyroid hormones (FTH) and total thyroid hormones (TTH) were insignificant; however, thyroid-stimulating hormone (TSH) levels were markedly elevated in patients with positive thyroid peroxidase antibody (TPOAb) and/or positive antithyroglobulin antibody (TgAb) (p<0.05). Additionally, gender disparities affected FTH levels (p<0.05) but not TTH and TSH levels. The female gender was significantly negatively correlated with serum Fe levels (r=-0.381, p<0.05). Serum Fe^3+^ deficiency also had an effect on FT3 in both genders, FT4 and TT4 in males (p<0.05), but not TSH (p>0.05). The multilinear regression model showed that TT3 (β=0.702), eGFR (β=0.109), Fe^3+^ (β=0.003), female gender (β=-0.061), and age (β=-0.061) were the major determinants for FT3 change. Moreover, renal function, which was represented as the estimated glomerular filtration rate (eGFR), had no effects on Fe^3+^ and TSH levels but on the levels of FTH and TTH (p<0.05). FT3/FT4 exhibited correlations with Fe^3+^ (r=0.252) and eGFR (r=0.285). Finally, changes in Fe^3+^ levels had no significant impact on fasting plasma glucose (FPG), fasting C-peptide, HbA1c, and glycated albumin levels (p>0.05).

***Conclusion*:** In addition to age, gender, and renal functions, serum Fe^3+^ levels in T2DM patients have a significant relationship with thyroid functions.

## 1 INTRODUCTION

Thyroid hormones are essential for cell proliferation, differentiation, and proper metabolism, growth, and development [1]. Maintenance of normal thyroid hormone levels depends on a range of trace elements required for thyroid hormone synthesis and metabolism, particularly iodine, selenium, copper, Fe^3+^, and zinc [2]. While the effects of iodine and selenium on thyroid hormones have been fully elucidated, the effects of other trace elements, including Fe^3+^, on thyroid hormone metabolism and production are yet to be conclusively established. Globally, Fe^3+^ deficiency is a serious health concern, impacting around 2 billion people [3]. Fe^3+^ deficiency affects iodine metabolism, particularly the process of iodine transformation to thyroxin [4].

Diabetes mellitus (DM) is one of the most prevalent diseases, and the global diabetes prevalence in 20-79 years olds in 2021 was estimated to be 10.5% (536.6 million people). This proportion is projected to rise to 12.2% (783.2 million) in 2045 [5]. It is a multi-organ disease that impairs various organs. Alterations in markers related to bone metabolism and thyroid functions in DKA [6] have been reported previously, along with the thyroid of patients with Type 1 diabetes mellitus (T1DM) in whom the glycemic is generally stable [7]. About 15-30% of individuals with T1DM are complicated by Graves’ disease and Hashimoto's thyroiditis. The relationship between T2DM and thyroid illness has yet to be established. T2DM alters microbiota species [8], which alters the absorption of trace micronutrients, such as Fe^3+^ [9], thereby impacting iodine metabolism and thyroid functions [10]. The significance of trace elements on thyroid functions and the interactions between gut microbiota and thyroid functions have been reported [2, 11]. In this study, we investigated the association between serum Fe^3+^ levels, thyroid functions, and other variables in T2DM patients.

## 2 MATERIAL AND METHODS

### 2.1 Acquisition of in-Patient Data

Data on T2DM patients from 2018-2021 were obtained from the in-patient information system of Shanghai Pudong Hospital. Patients with T1DM, hypothyroidism, and hyperthyroidism were excluded. Patients with extreme conditions, including severe cardiovascular and cerebral diseases, diabetic ketoacidosis, hyperglycemic hyperosmolar state, and severe infections, were also excluded. T2DM diagnosis was performed using the World Health Organization (WHO) guidelines published in 1999. The flowchart for study population selection is shown in Fig. (**1**).

### 2.2 Blood Sampling and Evaluation of Laboratory Data

We included laboratory data on diabetes status, thyroid functions, and trace micronutrients. After blood sampling, all parameters, including fasting blood glucose, c-peptide, HbA1C, eGFR, creatine, TSH, FT3, FT4, TT3, TT4, TPOAb, TgAb, serum Fe^3+^, serum calcium, serum magnesium and phosphonium ion levels were evaluated. All patients' thyroid hormone levels were within the euthyroid range, and there was no evidence of hyperthyroidism or hypothyroidism-related symptoms, such as sweating, changes in appetite, an increase in heart rate, or digestive dysfunction.

### 2.3 Statistical Analyses

Analyses were performed using SPSS (IBM, version 26.0) and GraphPad Prism version 9.0 software. Non-parametric analyses or two-way ANOVA were used to compare differences in levels of plasma C-peptide, HbA1c, serum Fe^3+^, and thyroid functions between and among groups. For the multiple testings, the method of Bonferroni corrections was adopted. Spearman correlation analyses were used to determine the relationships between gender disparity, thyroid functions, FT3/FT4, eGFR, and serum Fe^3+^ levels. Multilinear regression analyses were used to develop models for best predictors of thyroid hormone FT3 changes. p≤0.05 was the threshold for significance.

## 3 RESULTS

Baseline characteristics of serum Fe^3+^, thyroid function, and other variables for type 2 diabetes mellitus patients

Apart from electrolytes, such as Na^+^, K^+^, Cl^-^, Ca^2+^, plasma albumin and globulin levels, baseline characteristics for our study participants are presented in Table **1**.

Besides TSH, differences in various parameters between patients with Hashimoto thyroiditis (HT) and those without were insignificant.

**Fig. (2):**
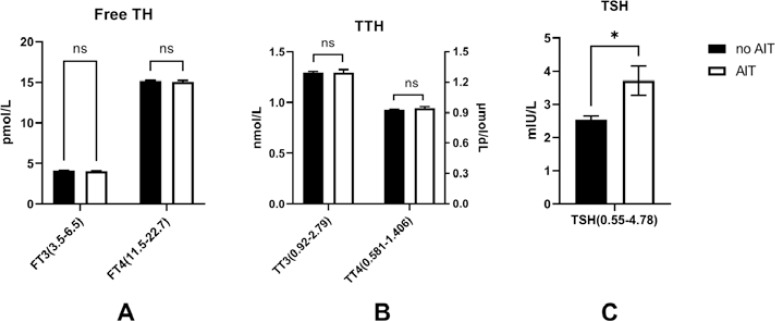
Thyroid functions between T2DM patients with or without autoimmune thyroiditis (AIT). Only TSH was significantly elevated in T2DM patients with AIT compared to those without (p<0.05). **Note:** *: p<0.05; ns: no significance; FTH: free thyroid hormones; TTH, total thyroid hormones; TSH: thyroid-stimulating hormone; AIT: autoimmune thyroiditis. The bar of each figure is displayed as Mean ± SEM (standard error).

First, we eliminated the possibility that autoimmune thyroiditis (AIT), such as Hashimoto thyroiditis, may affect thyroid functions. These diseases were classified based on the presence of positive TPOAb and/or TgAb (TPOAb>60 U/ml, TgAb>60 U/ml, reference range: TPOAb: 0~60U/mL; TgAb: 0~60U/mL). Apart from TSH (no AIT *vs.* AIT: 2.54 ± 3.92 mIU/L *vs.* 3.71 ± 6.84 mIU/L, p<0.05; reference range: 0.55~4.78mIU/L), differences in free thyroid hormones (free TH) (free T3: no AIT *vs.* AIT: 4.13 ± 0.89 pmol/L *vs.* 4.05 ± 1.08 pmol/L, p>0.05, reference range: 3.5~6.5pmol/L; free T4: no AIT *vs.* AIT: 15.19 ± 2.42 pmol/L *vs.* 15.07 ± 2.77 pmol/L, p>0.05, reference range: 11.50~22.70p mol/L) and total thyroid hormones (TTH) (total T3: no AIT *vs.* AIT: 1.30 ± 0.36 nmol/L *vs.* 1.30 ± 0.39 nmol/L, p>0.05, reference range: 0.92~2.79 nmol/L; total T4: no AIT *vs.* AIT: 92.75 ± 20.45 nmol/L *vs.* 94.31 ± 21.34 nmol/L, p>0.05, reference range: 58.1~140.6 nmol/L) between the groups were insignificant (Fig. **2**).

### 3.1 Gender is a Significant Factor in Thyroid Functions

**Fig. (3):**
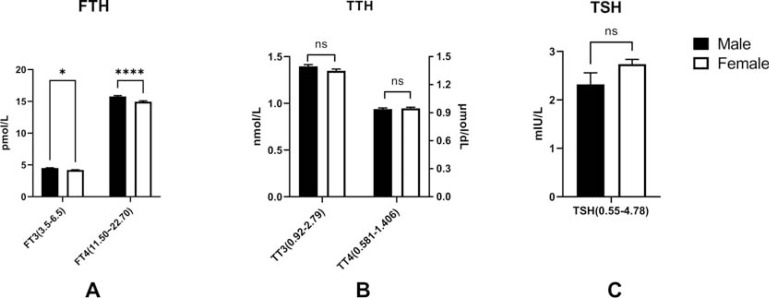
The implications of gender variation on thyroid function in T2DM patients without AIT. Both free T3 (p<0.05) and free T4 (p<0.0001) were markedly low in female T2DM patients. **Note:** *: p<0.05; ****: p<0.0001; ns: no significance; The bar of the each figure is displayed as Mean ± SEM (standard error).

We evaluated the possibility of gender differences with regard to thyroid functions in T2DM patients. After excluding AIT patients, there was a significant decrease in free thyroid hormone levels among female patients (FT3: male *vs.* female: 4.52 ± 0.82 pmol/l *vs.* 4.20 ± 0.74 pmol/l, p<0.05, reference range: 3.5~6.5pmol/L; FT4: male *vs.* female: 15.78 ± 2.42 pmol/l *vs.* 14.97 ± 2.24 pmol/l, p<0.0001, reference range: 11.50~22.70pmol/L), but not in total thyroid hormones and TSH (total T3: male *vs.* female: 1.40 ± 0.33 nmol/l *vs.* 1.35 ± 0.33 nmol/l, reference range: 0.92~2.79 nmol/L; total T4: male *vs.* female: 93.97 ± 19.15 nmol/l *vs.* 94.75 ± 19.98 nmol/l; p>0.05, reference range: 58.1~140.6 nmol/L) and TSH (male *vs.* female: 2.32 ± 4.26 mIU/L *vs.* 2.74 ± 1.76 mIU/L; p>0.05, reference range: 0.55~4.78 mIU/L) (Fig. **3**).

We determined whether the female gender also had an effect on serum Fe^3+^ levels, which may have affected thyroid functions in our study. Association analyses confirmed that gender disparity was significantly negatively correlated with FT3, FT4, and Fe^3+^ levels but positively correlated with TSH levels among females. These findings indicated that compared to males, female T2DM patients were more likely to have low FT3, FT4, and serum Fe^3+^ levels and higher TSH levels (Table **2**).

### 3.2 Differences in Serum Fe^3+^ Levels Affected Thyroid Functions in Both Male and Female T2DM Patients

**Fig. (4):**
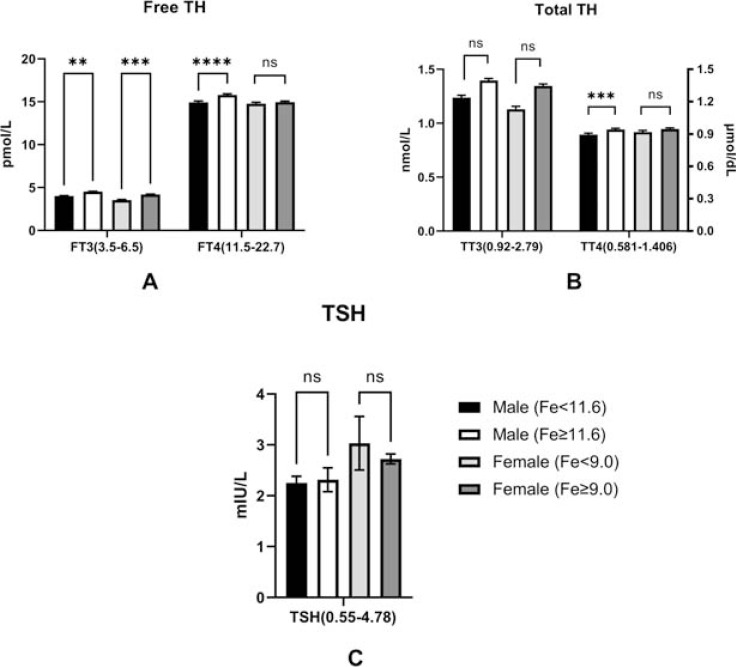
The comparisons of thyroid functions in the presence or absence of Fe^3+^ deficiency. Fe^3+^ deficiency had an effect on (**A**) FT3 levels in both male and female T2DM patients, FT4 levels, and TT4 levels only in males (**B**). (**C**). Fe^3+^ deficiency had no effects on TSH levels in both genders. **Note:** *: p<0.05; **: p<0.01; ***: p<0.001; ****: p<0.0001; ns: no significance. The bar of each figure is displayed as Mean ± SEM (standard error); Fe: Fe^3+^

We conducted a stepwise analysis to determine whether Fe^3+^deficiency or overabundance had any effects on thyroid functions. It was found that Fe^3+^ deficiency had significant effects on FT3 levels in both male and female patients (male: Fe^3+^<11.6 *vs.* ≥11.6: 4.00 ± 0.91 pmol/l *vs.* 4.52 ± 0.82 pmol/l; p<0.01; female: Fe^3+^<9.0 *vs.* ≥9.0: 3.55±0.85 pmol/l *vs.* 4.20 ± 0.74 pmol/l; p<0.001; reference range: 3.5~6.5pmol/L); FT4 in males only (male: Fe^3+^<11.6 *vs.* ≥11.6: 14.93 ± 2.54 pmol/l *vs.* 15.80 ± 2.41 pmol/l; p<0.0001; females: Fe^3+^<9.0 *vs.* ≥9.0: 14.77 ± 2.28 pmol/l *vs.* 14.95 ± 2.26 pmol/l; p>0.05; reference range: 11.50~22.70 pmol/L), TT4 in males only (male: Fe^3+^<11.6 *vs.* ≥ 11.6: 89.31 ± 21.29 nmol/l *vs.* 94.20 ± 19.41 nmol/l; p<0.05; females: serum Fe^3+^<9.0 *vs.* ≥9.0: 91.82 ± 20.74 nmol/l *vs.* 94.57 ± 20.07 nmol/l; p>0.05, reference range: 58.1~140.6 nmol/L). The differences in TT3 (male: Fe^3+^<11.6 *vs.* ≥11.6: 1.24 ± 0.34 nmol/l *vs.* 1.40 ± 0.33 nmol/l; p>0.05; female: Fe^3+^<9.0 *vs.* ≥9.0: 1.13 ± 0.37 nmol/l *vs.* 1.35 ± 0.33 nmol/l; p>0.05, reference range: 0.92~2.79 nmol/L), TSH (male: Fe^3+^<11.6 *vs.* ≥11.6: 2.25 ± 1.95 mIU/L *vs.* 2.31 ± 4.23 mIU/L; p>0.05; female: Fe^3+^<9.0 *vs.* ≥9.0: 3.03 ± 7.14 mIU/L *vs.* 2.72 ± 1.76 mIU/L; p>0.05, reference range: 0.55~4.78mIU/L) between male and female were not significant (Fig. **4**).

### 3.3 The Multilinear Regression Analyses Reveal Serum Fe^3+^ was a Critical Determinant of the Thyroid Function

After adjusting for age, gender, thyroid hormones, Fe^3+^, kidney functions, fasting plasma glucose levels, fasting C-peptide, and HbA1c levels in patients, we established a multilinear regression analyses model that revealed TT3, eGFR, Fe, Gender, and age to be significant determinants of FT3 levels (Table **3**).

### 3.4 Renal Functions were Associated with Thyroid Dysfunctions, Independently of serum Fe^3+^ Levels

**Fig. (5):**
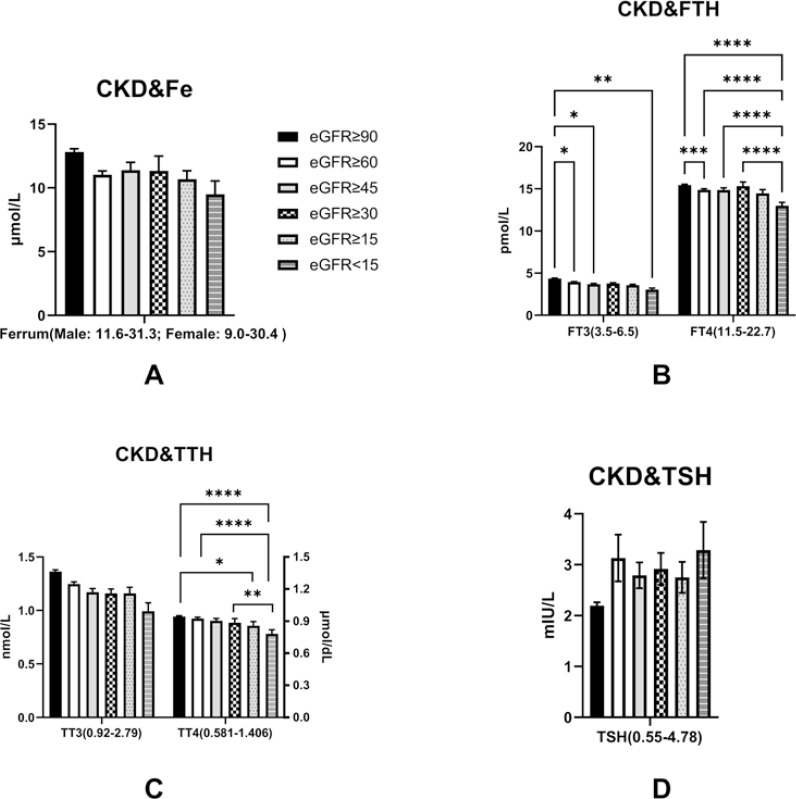
The comparisons of thyroid function at various stages of chronic kidney disease represented by eGFR levels. **Note:** *: p<0.05; **: p<0.01; ***: p<0.001; ****: p<0.0001; The bar of the each figure is displayed as Mean ± SEM (standard error). Fe: Fe^3+^

Since eGFR was established to be an independent predictor for FT3 levels, we assessed the effects of CKD on Fe^3+^ and thyroid functions. We established that Fe^3+^ levels were generally comparable across all eGFR stages (eGFR≥90: 12.82±5.51 μmol/l; eGFR≥60: 11.03±4.86 μmol/l; eGFR≥45: 11.39±5.50 μmol/l; eGFR≥30: 11.33±7.26 μmol/l; eGFR≥15: 10.68±3.38 μmol/l; eGFR<15: 9.50±5.05 μmol/l; p>0.05; reference range: Fe^3+^: Male: 11.6-31.3mmol/L, female: 9.0-30.4mmol/L), while thyroid hormones, including FT3, FT4, TT3, and TT4 exhibited gradual decline at each eGFR level (FT3: eGFR≥90: 4.36±0.91 pmol/l; eGFR≥60: 3.95±0.79 pmol/l; eGFR≥45: 3.69±0.79 pmol/l; eGFR≥30: 3.77±0.56 pmol/l; eGFR≥15: 3.57±0.55 pmol/l; eGFR<15: 3.06±0.81 pmol/, reference range: FT3: 3.5-6.5 pmol/L; FT4: eGFR≥90: 15.44±2.34 pmol/l; eGFR≥60: 14.87±2.31 pmol/l; eGFR≥45: 14.86±2.31 pmol/l; eGFR≥30: 15.33±3.06 pmol/l; eGFR≥15: 14.45±2.34 pmol/l; eGFR<15: 12.99±1.95 pmol/l, reference range: FT4: 11.5-22.7pmol/L; TT3: eGFR≥90: 1.36±0.36 nmol/l; eGFR≥60: 1.25±0.33 nmol/l; eGFR≥45: 1.17±0.31 nmol/l; eGFR≥30: 1.16±0.27 nmol/l; eGFR≥15: 1.16±0.30 nmol/l; eGFR<15: 0.99±0.37 nmol/l, reference range: TT3: 0.92-2.79nmol/L; TT4: eGFR≥90: 94.27±19.13 nmol/l; eGFR≥60: 92.413±21.37 nmol/l; eGFR≥45: 90.28±21.43 nmol/l; eGFR≥30: 88.56±24.94 nmol/l; eGFR≥15: 85.67±20.28 nmol/l; eGFR<15: 78.21±17.69 nmol/l, reference range: TT4: 58.1-140.6nmol/L). Low eGFR levels had no effects on TSH levels (eGFR≥90: 2.20±1,51 mIU/L; eGFR≥60: 3.13±7.47 mIU/L; eGFR≥45: 2.79±2.38 mIU/L; eGFR≥30: 2.92±2.04 mIU/L; eGFR≥15: 2.75±1.59 mIU/L; eGFR<15: 3.29±2.71 mIU/L; p>0.05, reference range:TSH: 0.55-4.78 mIU/L) (Fig. **5**).

Variations in eGFR levels showed no significant effects on serum Fe^3+^ levels (A); FT3 and FT4 levels showed a significantly decreased trend in response to the reduction in eGFR levels (B) TT4 levels exhibited decreasing tendencies as eGFR levels decreased (C); TSH levels were comparable in the group of each eGFR stage (E). The multiple comparisons of thyroid function among each eGFR stage using the method of Bonferroni corrections.

### 3.5 Association of Thyroid Hormone Bioactivity with Serum Fe^3+^ Levels

Moreover, the FT3 to FT4 ratio (FT3/FT4), an indicator of the efficacy of FT4 to FT3 conversion and thyroid hormone bioactivity, was significantly associated with serum Fe^3+^ levels (Table **4**).

### 3.6 Effects of Serum Fe^3+^ Levels on Glucose Metabolism in T2DM Patients

**Fig. (6):**
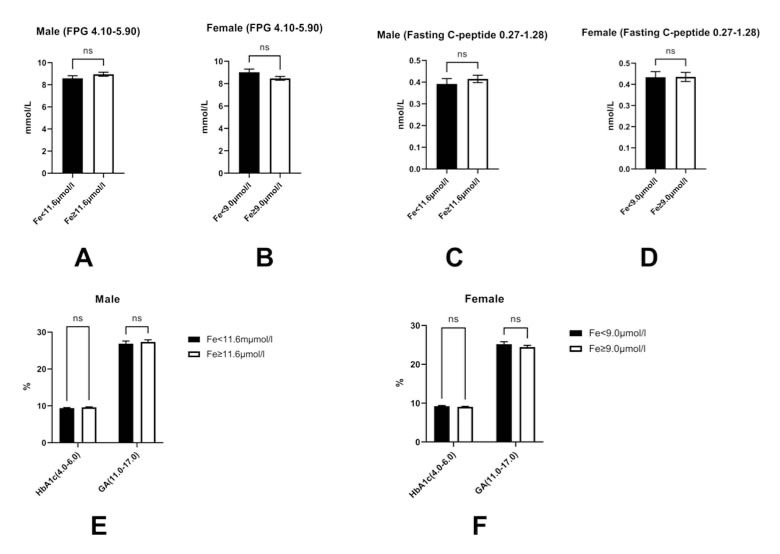
Effects of serum Fe^3+^ levels on glucose metabolism-related indices.

Finally, we investigated the effects of serum Fe^3+^ levels on glucose metabolism. Serum Fe^3+^ deficiency had no significant effects on glucose metabolic markers, including fasting plasma glucose (FPG) levels (male: Fe^3+^<11.6 μmol/l *vs.* Fe^3+^≥11.6 μmol/l: 8.58±3.79 mmol/l *vs.* 8.95±3.55 mmol/l; p>0.05; female: Fe^3+^<9.0 μmol/l *vs.* Fe^3+^≥9.0 μmol/l: 9.02±4.19 mmol/l *vs.* 8.47±3.49 mmol/l; p>0.05, referece range: 4.10~5.90 mmol/L), fasting C-peptide levels (male: Fe^3+^<11.6 μmol/l *vs.* Fe^3+^≥11.6 μmol/l: 0.39±0.37 nmol/l *vs.* 0.42±0.32 nmol/l; p>0.05; female: Fe^3+^<9.0 μmol/l *vs.* Fe^3+^≥9.0 μmol/l: 0.43±0.37 nmol/l *vs.* 0.44±0.43 nmol/l; p>0.05, reference range: 0.27~1.28 nmol/L), HbA1c levels (male: Fe^3+^<11.6 μmol/l *vs.* Fe^3+^≥11.6 μmol/l: 9.38±2.48% *vs.* 9.62±2.37%; p>0.05; female: Fe^3+^<9.0 μmol/l *vs.* Fe^3+^≥9.0 μmol/l: 9.24±2.33% *vs.* 9.06±2.16%; p>0.05, reference range: 4.0~6.0%), glycated albumin (GA) levels (male: Fe^3+^<11.6 μmol/l *vs.* Fe^3+^≥11.6 μmol/l: 26.87±11.02% *vs.* 27.33±10.75%; p>0.05; female: Fe^3+^<9.0 μmol/l *vs.* Fe^3+^≥9.0 μmol/l: 25.19±9.31% *vs.* 24.43±8.90%; p>0.05, reference range: 11.0~17.0%) (Fig. **6**).

Fe^3+^ deficiency had no significant effects on glucose metabolism indicators in T2DM patients, including FPG (A and B), fasting C-peptide (C and D), HbA1c, and GA (E and F). Note: ns: no significance; FPG: fasting plasma glucose; HbA1c: hemoglobulin A1c; GA: glycated albumin. Fe: Fe^3+^. The bar of each figure is displayed as Mean±SEM (standard error).

## 4 DISCUSSION

Patients with diabetes mellitus (DM) are at risk of developing thyroid abnormalities or illnesses [12]. Due to the autoimmune and genetic etiology of type 1 diabetes mellitus (T1DM), there is a greater predisposition for various complications, such as Hashimoto thyroiditis [13]. This thyroid disorder is highly common in T2DM patients compared to the general population [14]. Thyroid disorders, such as hyperthyroidism or hypothyroidism, can centrally or peripherally worsen glucose metabolism, resulting in insulin resistance or diabetic cardiovascular complications [15]. For instance, blood leptin levels are correlated with TSH levels and are elevated in hypothyroidism patients; leptin stimulates TSH synthesis by affecting the hypothalamus-pituitary-thyroid (HPT) axis *via* the Janus activating kinase (JAK)-2/signal transducer and activator of transcription 3 (STAT3) factor. Autoimmune thyroiditis (AIT) and the female gender may be risk factors for thyroid disorders. We assessed the potential risk factors for thyroid dysfunction in T2DM patients in this study. It was established that serum Fe^3+^ levels were significantly associated with thyroid functions but had no marked effects on glucose metabolism. Our findings indicate that serum Fe^3+^ levels in T2DM patients modulate thyroid functions and that Fe^3+^ supplementation may promote the maintenance of thyroid homeostasis in T2DM.

In our preliminary analyses, we compared the presence of AIT to variations in thyroid functions. It was found that autoimmune thyroid disease was frequent in T2DM patients. Additionally, an association between Fe^3+^ deficiency and autoimmune thyroid illnesses has been reported [16]. Patients with AIT are usually Fe^3+^ deficient, as autoimmune gastritis and celiac disease frequently occur in AIT patients to inhibit Fe^3+^ absorption [2]. Since Fe^3+^ affects thyroid hormone synthesis and transformation, we found that Fe^3+^ can affect FTH and TTH levels in T2DM patients.

In this study, it was also established that gender disparity influenced FTH and was substantially associated with Fe^3+^ levels. Gender disparity only affected the blood level of FT3 and FT4. This could be attributed to differences in hormones and physiology between T2DM male and female patients, which predisposed females to AIT and other thyroid illnesses [17]. Various factors may contribute to disparities in serum Fe^3+^ levels between men and women. For instance, gender differences are associated with altered interactions between muscle and adipose tissues with thyroid hormones [18]. In this study, serum Fe^3+^ deficiencies were correlated with low FT3, FT4, and TT4 levels in males but only FT3 in females [19]. Then, we established a multilinear regression to predict FT3 changes, which was consistent with findings from previous studies. Among the indices, TT3 is closely and dynamically associated with FT3 transformation; eGFR has been associated with thyroid functions, particularly thyroid sensitivity; age is another key element that influences FT3 levels, as it affects the H-P-T axis [20-22]. All indices in this study were independent variables for FT3 level changes, which could explain 62.8% (R square) of the FT3 change, representing active thyroid functions. Additionally, the FT3/FT4 ratio, which indicattes the efficacy of FT4 to FT3 conversion and thyroid hormone bioactivity, was significantly associated with serum Fe^3+^ levels, implying that these independent variables are both related to thyroid hormone conversion and they act peripherally. Finally, we investigated the effects of varying serum Fe^3+^ levels on glucose metabolism markers. We established that none of the glucose metabolism indices were associated with serum Fe^3+^ levels.

**Fig. (7):**
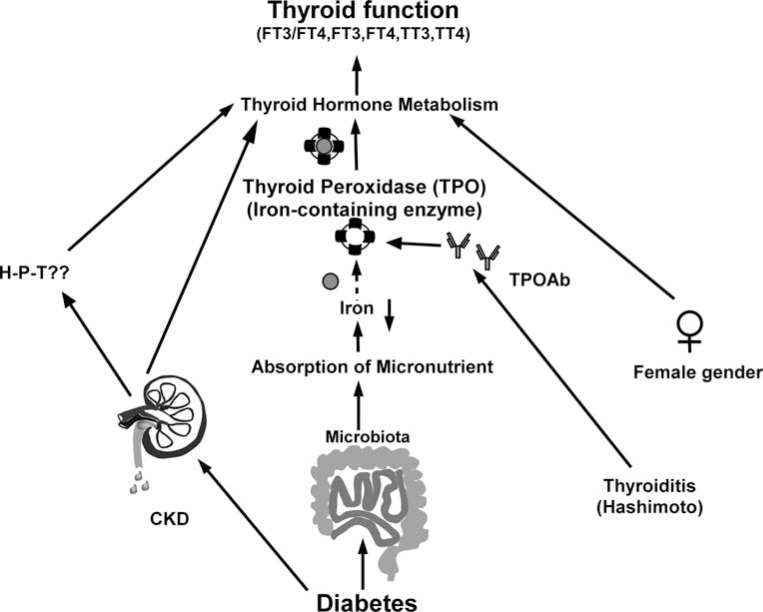
Potential key factors that contribute to thyroid dysfunction in T2DM patients include chronic kidney disease, iron (Fe^3+^) deficiency, thyroiditis, and gender. In T2DM patients, it is possible that the microbiota composition affects the absorption of micronutrients, such as iron, a critical co-enzyme for thyroid peroxidase (TPO), which is required for thyroid hormone metabolism. Impaired renal function may have an effect on both the HPT axis and thyroid function *via* an unknown pathway. The autoimmune process in thyroiditis, such as Hashimoto's disease (HT), can affect TPO enzyme functions and thyroid hormone metabolism. Another significant factor is the female gender.

Various trace micronutrients, including iodine, selenium, copper, and Fe^3+,^ have been implicated in thyroid metabolism and functions [23]. Fe has a marked effect on iodine metabolism, particularly during iodine transformation to thyroxine. Fe^3+^ deficiency has the following effects on thyroid metabolism: 1. Fe^3+^ deficiency impairs thyroid metabolism by dysregulating iodine activation, which is catalyzed by thyroid peroxidase. The thyroid peroxidase active metabolic center is the heme (Fe^3+^-containing) enzyme. Thyroid peroxidase, like all Fe^3+^-containing enzymes, is extremely sensitive to Fe^3+^ ingestion. Fe^3+^ deficiency results in decreased thyroid peroxidase activities, which affects thyroid hormone synthesis. 2) Fe^3+^ is a cofactor for various enzymes involved in several physiological functions and metabolism, including the tricarboxylic acid (ATP production) cycle. When Fe^3+^ levels are low, both Fe^3+^-containing and Fe^3+^-dependent enzymes exhibit decreased activities. Inhibited adenosine triphosphate (ATP) synthesis and energy reduction impair the functions of thyroid follicles, resulting in decreased thyroid hormone synthesis. Second, superoxide dismutase activities regulate thyroid hormone production. 3) Fe^3+^ deficiency decreases the activities of thyroxine 5 deiodinase in the liver, inhibits the conversion of peripheral FT4 to FT3, and decreases thyroid hormone levels in plasma, particularly FT3 [2, 24]. Any or all of the mechanisms described above regarding the effects of Fe^3+^ on thyroid metabolism may be impaired in T2DM patients (Fig. **7**). We found that Fe^3+^ deficiency is consistently associated with thyroid functions in T2DM patients, indicating that Fe^3+^ levels are an important regulator of thyroid functions.

In this study, we did not analyze liver enzymes, serum albumin, serum globulin, or other micronutrients, such as selenium or copper, which are all necessary for thyroid hormone functions. This is because they have been well studied in earlier investigations. Due to the complexity of T2DM patients, we identified multiple critical parameters associated with changes in thyroid functions; thyroid functions may also be impacted by other factors in these individuals. Thus, more studies should also be performed to elucidate the diabetes-iron-thyroid association.

In this study, we explored the relationship between serum iron and thyroid function, which could be a critical link and explanation for the thyroid function changes in T2DM, and summarized the possible factors that influence the thyroid function status in T2DM. As per our knowledge, this is the first study that reveals this relationship, and the analysis partially suggests the implication of iron supplemention in T2DM. However, our study results were limited by some conditions, such as sample size, single-center study, and normal population without T2DM. We believe in future studies, we will address and deeply investigate such issues and incorporate them into our analyses.

## CONCLUSION

Among the various factors affecting thyroid hormone metabolism in T2DM patients, such as gender disparity, chronic kidney disease, and thyroiditis, serum Fe^3+^ is a critical factor required for thyroid hormone metabolism, and its level affects TPO activities, which are involved in the regulation of thyroid hormone activities, particularly FT3 activities. Iron deficiency medication may potentially benefit these patients' thyroid functions.

## Figures and Tables

**Fig. (1):**
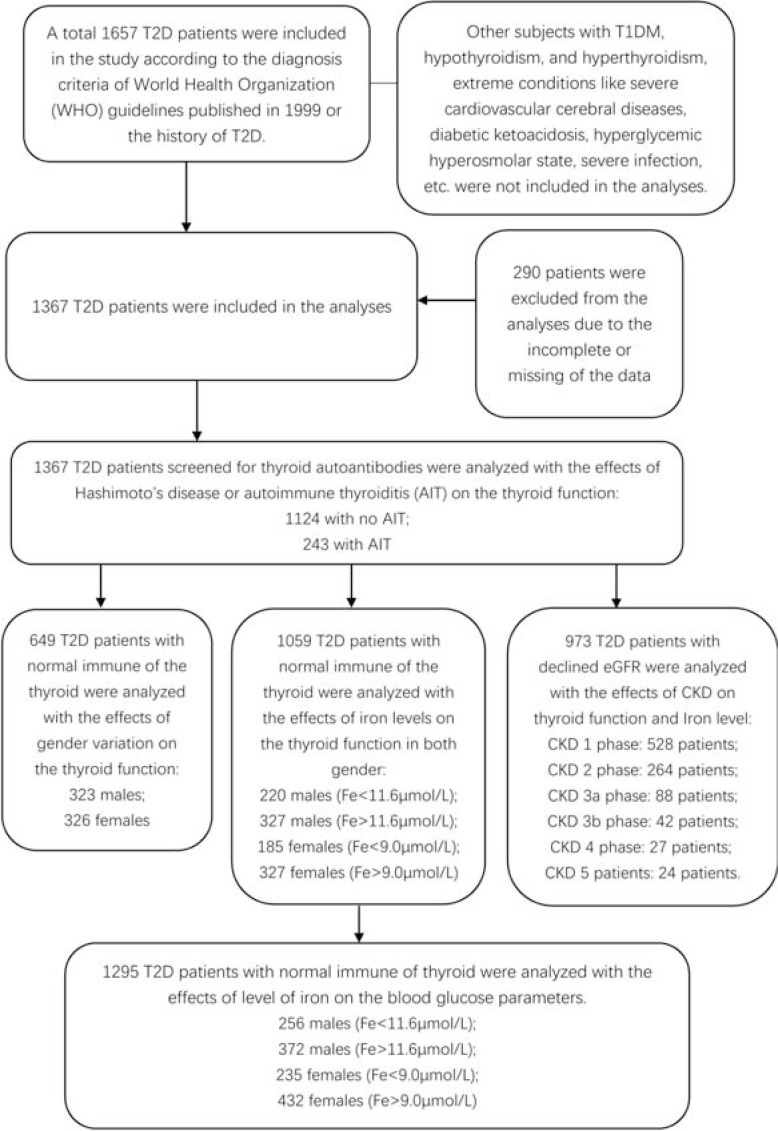
The flowchart for patient selection and data analyses in this study. **Note:** Fe: Fe^3+^

**Table 1:** Baseline characteristics for type 2 diabetes mellitus patients.

**Variables**	**Values**	**Reference Ranges**
N (M/F)	1657(815/842)	/
Age (year)	64.40 ± 13.91	/
BMI (kg/m^2^)	26.14 ± 8.94	18-24
HbA1c (%)	9.27 ± 2.33	4.0-6.0%
Duration of diabetes (y) eGFR (ml/min/1.73m^2^) Creatinine (μmol/L) TT3 (nmol/L) TT4 (nmol/L) FT3 (pmol/L) FT4 (pmol/L) TSH (mIU/L) TPOAb (U/ml) TgAb (U/ml) Fe^3+^ (μmol/L) Ca^2+^ (mmol/L)	10.14 ±7.77 85.43 ± 28.69 91.92 ± 101.75 1.30 ± 0.36 92.95 ± 20.57 4.11 ± 0.93 15.17 ± 2.48 2.75 ± 4.59 90.74 ± 174.02 49.75 ± 78.59 11.96 ± 5.36 2.18 ± 0.14	/ CKD-EPI calculation 41-73 0.92-2.79 58.1-140.6 3.5-6.5 11.5-22.7 0.55-4.78 0-60 0-60 Male: 11.6-31.3 Female: 9.0-30.4 2.08-2.65

**Note:** The values were presented as mean ± standard error.

**Table 2:** Correlations between gender, thyroid hormones, and Fe levels.

-	**TT3**	**TT4**	**FT3**	**FT4**	**TSH**	**Fe^3+^**
**Correlations with Gender Disparity (r)**	-0.037	-0.094	-0.283	-0.209	0.239	-0.381
**Significance (p)**	0.707	0.332	0.003	0.028	0.012	<0.001

**Table 3:** Multi-linear regression model of variables related to FT3 levels.

**Multi-linear Regression**	**R**	**0.792**	**R^2^**	**0.628**	**Adjusted R^2^**	**0.626**	**-**	**-**
-	Variables	B	SE	β	t	Sig	Tolerance	VIF
-	Constant	1.547	0.120		12.925	<0.001	-	-
	TT3	1.867	0.042	0.702	44.694	<0.001	0.914	1.094
	eGFR	0.004	0.001	0.109	6.369	<0.001	0.775	1.291
	Fe^3+^ Gender age	0.019 -0.103 -0.004	0.003 0.026 0.001	0.111 -0.061 -0.061	6.983 -3.936 -3.529	<0.001 <0.001 <0.001	0.892 0.936 0.754	1.121 1.068 1.327

**Note:** SE: stand error; Sig: significance. VIF: variance inflation factor.

**Table 4:** Correlation relationship between FT3/FT4 ratio with serum Fe^3+^ levels and eGFR stage.

**-**	**Fe^3+^**	**eGFR**
Correlation with FT3/FT4 (r)	0.252	0.285
Significance (p)	<0.001	<0.001

## Data Availability

All data generated or analyzed during this study are included in this published article.

## LIST OF ABBREVIATIONS

FTH: Free Thyroid Hormones
T2DM: Type 2 Diabetes Mellitus
TPOAb: Thyroid Peroxidase Antibody
TSH: Thyroid-Stimulating Hormone
TTH: Total Thyroid Hormones

## STANDARDS OF REPORTING

STROBE guidelines were followed in this study.
